# Experience of 1166 Thyroidectomy without Use of Prophylactic Antibiotic

**DOI:** 10.1155/2014/758432

**Published:** 2014-05-12

**Authors:** Qiang Lu, Shu-Qin Xie, Si-Yuan Chen, Li-Ju Chen, Qian Qin

**Affiliations:** ^1^Department of General Surgery, TungWah Affiliated Hospital of Sun Yat-sen University, Dongguan, Guangdong 523110, China; ^2^Department of Surgical Oncology, Tung Wah Affiliated Hospital of Sun Yat-sen University, Dongguan, Guangdong 523110, China; ^3^Operating Room Tung Wah, Tung Wah Affiliated Hospital of Sun Yat-sen University, Dongguan, Guangdong 523110, China

## Abstract

*Background*. Although the procedure requires a small surgical incision and a short duration, incision infection rate is very low in thyroidectomy; however, doctors still have misgivings about infection events. *Aim*. We retrospectively analyzed the prevention of incision infection without perioperative use of antibacterial medications following thyroidectomy. *Materials and Methods*. 1166 patients of thyroidectomy were not administered perioperative antibiotics. Unilateral total lobectomy or partial thyroidectomy was performed in 68.0% patients with single-side nodular goiter or thyroid adenoma. Bilateral partial thyroidectomy was performed in 25.5% patients with nodular goiter or Graves' disease. The mean time of operation was 80.6 ± 4.87 (range: 25–390) min. *Results.* Resuturing was performed in two patients of secondary hemorrhage from residual thyroid following bilateral partial thyroidectomy. Temporally recurrent nerve paralysis was reported following right-side total lobectomy and left-side subtotal lobectomy in a nodular goiter patient. One case had suppurative infection in neck incision 5 days after bilateral partial thyroidectomy. *Conclusions*. Thyroidectomy, which is a clean incision, involves a small incision, short duration, and minor hemorrhage. If the operation is performed under strict conditions of sterility and hemostasis, antibacterial medications may not be required to prevent incision infection, which reduces cost and discourages the excessive use of antibiotics.

## 1. Introduction

Thyroidectomy is a common general surgery procedure involving a clean type-I incision. Although the procedure requires a small surgical incision and a short duration, most hospitals in China are still using antibacterial medications perioperatively in order to prevent the incision infection. In this regard, first, second and even third generation cephalosporins were commonly used. Cephalosporins were also used together with other antibacterial medications. Some cases involved in serious adverse effects when use of quinolones and aminoglycosides.

As for type-I incision, the preventative use of antibacterial medications was intended mainly to kill or inhibit the contaminating bacteria from air, environment, or patient. However, more and more evidence suggests that it may not be necessary to use antibacterial medications to prevent the incision infection [1–11]. Therefore, unreasonable use of prophylactic antibiotic will not only be uneconomical but will also involve risks in the development of multiple drug resistance in bacteria. The development of multiple drug resistance in bacteria due to excessive use of antibacterial medications is a major cause of the failure of therapy in many prevailing human infections. In order to prevent the newly emerging hard-to-treat infection, a great deal of caution is warranted for the use of antibacterial medications by physicians.

In this study, we have included data from 1166 patients that underwent thyroidectomy at our hospital from January 2008 to February 2014. All the patients were not routinely given antibacterial medications for prevention of incision infection and good outcome was achieved.

## 2. Materials and Methods

### 2.1. Patients

Between January 2008 to February 2014, 1166 patients of thyroidectomy in our hospital were not administered perioperative antibiotics for the prevention of incision infection. Of the 1166 cases, 212 were male and 954 were female. The median age was 37.6 ± 6.14 (range: 10–81) years. With regard to age groups, 29 patients were 11–20 years old, 256 patients were 21–30 years old, 372 patients were 31–40 years old, 233 patients were 41–50 years old, 208 patients were 51–60 years old, 60 patients were 61–70 years old, and 9 patients were 71–80 years old. The patients' characteristics are outlined in Table 1.

### 2.2. Tumor Pathotypes

In regard to tumor types (Table 1), there were 916 cases of nodular goiter, 114 cases of thyroid adenoma (70 cases of follicular adenoma and 44 cases of thyroid papillary adenoma), 45 cases of Graves' disease, 71 cases of papillary carcinoma, 14 cases of Hashimoto's disease, 2 cases of medullary carcinoma, one case of leiomyosarcoma, and 4 cases of parathyroidoma.

### 2.3. Preoperative Disease Problems

As summarized in Table 1, preoperative health problems were observed in 66 patients that included 32 cases of diabetes mellitus, 34 cases of hypertension, and one case of infiltrative pulmonary tuberculosis which required bilateral partial thyroidectomy for nodular goiter; this operation lasted for 165 minutes.

### 2.4. Surgical Procedure and Postoperative Management

Cervical plexus anesthesia was performed in 163 patients and tracheal intubation general anesthesia was performed in 1003 patients. Sterilization with 0.5% iodophor or 2% iodine tincture was performed according to surgical requirement. For benign disease, cervical collar incision was made in 904 patients while endoscopic transaxillary or breast areola approach was used in 262 patients. Unilateral total or partial thyroid lobectomy was performed in 868 patients with single-side nodular goiter or thyroid adenoma while bilateral partial thyroidectomy was performed in 290 patients with nodular goiter or Graves' disease. In patients with Graves' disease, 6–8 g of thyroid body was generally saved. After bleeding on the trauma surface was checked, trauma lumen was rinsed with physiological saline and a drainage tube was placed inside trauma lumen. For simple single lobectomy or subtotal lobectomy, a drainage tube of 0.3 cm diameter was introduced; however, when the trauma lumen was too big, such as for double lobectomy in Graves' disease or in case of a surgical procedure involving malignant tumor, a drainage tube of larger diameter was placed for constant negative pressure drainage. Depending on surgeon's choice, drainage tube could be extracted via incision or through a small opening made at the posterior border of sternomastoid muscle of supraclavicular fossa. The latter was located far away from the site of incision, was more concealing, and it left only a minor scar and, therefore, this method was generally preferred. Resection of parathyroidoma was performed in 4 (0.34%) cases.

One patient with Grave's disease underwent second total resection of the residual lobe and the other Grave's disease patient underwent third total resection of the residual lobe (second surgical procedure was performed due to recurrence and third surgical procedure was performed due to complicated nodular goiter caused by residual thyroid).

L-type incision was performed in all 6 cases of malignant tumor. One patient underwent bilateral cervical lymph node resection (one side cervical lymph node resection was performed first and cervical lymph node resection was performed on the other side after 2 weeks), 3 cases underwent unilateral cervical lymph node resection, and one case underwent bilateral cervical lymph node resection at the same time. Unilateral cervical lymph node resection for papillary carcinoma was performed in 4 cases and bilateral cervical lymph node resection for papillary carcinoma was performed in one case. Resection of parathyroidoma was performed in 4 (0.34%) cases.

Median time of surgery was 80.6 ± 4.87 (range: 25–390) min. Furthermore, out of the total 1166 surgical procedures performed, 418 operations lasted for 20–69 min, 658 operations lasted for 70–120 min, 65 operations lasted for 130–170 min, 18 operations lasted for 180–230 min, and 7 operations lasted for 240–330 min. The data concerning anesthesia, surgical procedure, and duration of surgery are shown in Table 2.

Drainage tube was removed at 36–48 hours postoperatively in patients with benign condition or those not requiring cervical lymph node resection. Regarding patients with a malignant condition or those requiring cervical lymph node resection, the drainage tube was removed at 1 week postoperatively.

## 3. Results

### 3.1. Diagnosis and Treatment

Benign nodular goiter was diagnosed at intraoperational pathological examination of fast-frozen tissue sections and 2 were diagnosed to have papillary carcinoma during routine pathological examination of the samples at 4 days postoperatively. Both cases underwent resection of the residual lobe and isthmus at 4 days after first surgery and resection of the opposite lobe at 6 days after first surgery.

### 3.2. Major Postoperative Complications

As listed in Table 2, major postoperative complications were observed in 10 patients. They were incision infection (one patient), bleeding after 24 hours of surgery (5 patients), laryngeal nerve paralysis (6 patients), hypoparathyroidism (2 patients), and allergic dermatitis exfoliativa caused by sodium phenobarbital (one patient). One patient who had right side total resection and left side subtotal resection developed recurrent laryngeal nerve paralysis and achieved full recovery in 4 months. Three patients had to undergo resuturing procedure to stop postoperative bleeding due to residual thyroid. Regarding one patient, blood was found in drainage tube after returning to ward from surgery and, therefore, resuturing was performed to stop the active hemorrhage from a small artery on the upper pole which was located as the incision suture was removed. The other patient underwent bilateral resection of the nodular goiter and lost about 130 mL blood within 3 hours after returning to ward and was, therefore, sent back to operating room to have the active hemorrhage of the left residual end resutured which was found after the incision suture was removed. One case (a 60-year-old female with nodular goiter) presented with postoperative complication of suppurative infection at neck incision. She underwent bilateral partial thyroidectomy in December 2007 under cervical plexus anesthesia and the operation lasted for 65 minutes. The skin around the incision was found to be inflamed at 5 days after the surgery. The patient's suture was removed for drainage and pus was cultured. The bacterium involved was identified as* Staphylococcus aureus epidermidis*. The patient was treated with intravenous injections of ceftriaxone sodium for 5 days and the incision heal was achieved within 14 postoperative days. Another case with nodular goiter reported allergic dermatitis exfoliativa caused by sodium phenobarbital and full recovery was achieved in 11 days. With regard to hospital stay and follow-up, the median hospital stay period was 5.2 (range: 3–14) days. All patients were followed up for 4-5 weeks after discharge from the hospital.

## 4. Discussion

As for type-I incision, prophylactic antibiotic intends mainly to kill or inhibit the contaminating bacteria from air, environment, or patient and thus to protect from an acquired infection (surgical incision infection, SSI). However, the excessive use of prophylactic antibiotic for prevention of infection is not only uneconomical but also involves potential risks of the development of multiple drug resistance in bacteria. This will eventually lead to the reduced effectiveness of available antibacterial drugs on the market. Based on current practice, most hospitals in China (including Grade-1 and Grade-3 hospitals) are using prophylactic antibiotic during the perioperational period of thyroid surgery. As opposed to strict regulatory policy guidance, the decision of the use of antibacterial regimens is left solely to treating surgeon's discretion and, therefore, some will choose to prescribe antibacterial regimens for pre-, intra-, and postoperative periods up until sutures are removed. Besides, many physicians opt for different drug combinations including second or third generation cephalosporins and sometimes even use antibacterial medications that may have adverse side effects, such as quinolones and aminoglycosides. The rationale for use of prophylactic antibiotic includes the following: (1) operating doctors have concerns about safety of operating rooms and postoperative infections due to environmental microbes; (2) a postoperative infection may lead to an argument between patient and doctors; (3) lack of a large-scale multicenter clinical trial study to evaluate if the preventive antibacterial medication during perioperative can be avoided in case of thyroid surgery; and (4) some clinicians may not be well aware of the potential risks arising from an excessive use of antibiotics.

We suggest that the pivotal strategy to prevent incision infection following thyroid surgery is to reinforce sterilization and validation of surgical staff and surgical instruments. Many studies have shown that with stringent sterilization measures put in place, the incidence rate of incision infection differed nonsignificantly between antibiotics-treated and untreated patients [12–17]. On the other hand, excessive use of prophylactic antibiotic during operation may sometimes make the attending personnel compromise on stringent sterilization and validation. As a standard of sterilization during thyroid surgery, important measures include sterilization of surgical field, protection of skin around the incision, and use of good/aseptic surgical technique and skills. Regarding choice of anesthesia, cervical plexus anesthesia sometimes does not induce full effect or even fails to induce an effect. If anesthesia is not properly induced, patients may develop headache or sometimes become restless, making it difficult for the surgeon to operate.

Movements during surgery may also cause displacement contamination of sterile surgical cloth placed on neck or even direct contamination of the wound if patient's hand touches trauma surface. One such incident took place as one of the patients included in this study had improper induction of cervical plexus anesthesia and she instantly touched the trauma surface during surgery, leading to incision infection. Culture identification revealed the involvement of* Staphylococcus aureus epidermidis*.

Thyroidectomy is a simple and short operation. However, if anesthesia does not work well, good anesthesia effect is hard to achieve even with additional analgesics or strong pain medications. As described above, we opine that tracheal intubation general anesthesia is by far the best option for thyroidectomy. With the development of general anesthesia techniques, thyroidectomy has become a very safe operation and patients can wake up soon after surgery. It does not add extra cost to the procedure as may be the case with cervical plexus anesthesia. Therefore, we induced general anesthesia in all thyroidectomy procedures since 2008. Based on the 3-year data from our hospital, we recommend it as first choice during thyroidectomy procedures.

Postoperative hemorrhage, complicated hematoma, and lymphadenopathy, are the common complications following thyroid surgery. Patients with mild condition are treated with drainage measures. If the drainage cannot be well performed, incision healing can be delayed due to postoperative complications and/or infection. For patients with a severe condition, the trachea may become pressed and cause blockage of respiratory tract which may lead to death without timely treatment. Therefore, hemostasis should be strictly performed as a basic measure to prevent complications. Nonetheless, the opinion about using drainage inside trauma lumen to prevent incision infection remains controversial [9–11, 16–18]. We still think that secondary operation or complicated hematoma will increase the chance of incision infection if hemostasis is not well performed or the drainage tube does not work well. Many random control clinical trial and retrospective studies [9–11, 16] showed that the placement of drainage might relate to incision infection. Tabaqchali et al. [11] analyzed data from a 14-year study and reported that the patients in drainage group had a significantly lower rate of incision infection as compared with those in the nondrainage group. However, another study indicated that there was no significant difference between these two groups [9]. In this retrospective study of 582 patients undergoing thyroid surgery [7], the incidence rate of incision infection without use of antibacterial medications was only 1%. Rosato et al. [4] analyzed data of 14934 cases undergoing thyroid surgery from 8 hospitals in Italy over 5 years; wound infection occurred in only 0.3% of patients. We, therefore, suggest that more attention to strict hemostasis and good drainage measures needs to be paid rather than emphasizing on preventative antibacterial therapy. Several factors, however, account for discrepancies found among findings of these two studies.

In regard with postoperative observation and treatment, general vital signs of patients as well as drainage should be observed for 24 hours after surgery. The drainage should be carefully monitored whether congestion and swelling are found in the neck incision/skin or surrounding area. Once complicated hematoma is formed and drainage cannot be established after adjustment, a blood clot will usually form and the patient will have to be sent back to operating room for clearing up of hematoma and repositioning of drainage tube. The most postoperative hemorrhages are located at the residual end of thyroid body or the cutting end of thyroid vessels. Hemostasis should be performed in operating room under general anesthesia to ensure the safety of operation and avoid incision infection. In this study, 3 cases underwent secondary operation due to hemorrhage from residual end of thyroid. However, none of the above patients suffered from incision infection without antibacterial medication before and after both surgical procedures. Due to the low incidence rate of incision infection following thyroid surgery, there was no significant difference found between patients on antibacterial medication and those who were kept off of medication [9, 15–18]. The existing data are inadequate to prove the importance of preventative use of antibacterial medications in such patients [16, 17]. A previous study [5] reported that the incidence rate of incision infection between thyroidectomy patients with and without antibacterial medications was 4.2% (2/48) and 6.9% (4/58), respectively, whereas another study [15] showed that these rates were 2.9% (2/67) and 0% (0/68), respectively. Thus, current data are inconclusive to suggest if the lower incidence rates of incision infection were due largely to the improvement of surgical techniques and skills, sterility measures, or application of broad spectrum antibacterials. However, based on results of the previous [9, 15–18] and this study, we suggest that the low incidence of incision infection in thyroid surgery may not be related to the use of antibacterial medications. Arguably, thyroid surgery involves type I incision which has small trauma surface, short operation time, and a clean skin location.

Therefore, under preconditions that patient does not have a basic disease and sterilization is followed strictly at all times during and after surgery, routine preventative antibacterial medication may be regarded as excessive. We rather suggest that the preventative use of antibacterials should only be considered if (1) the patient needs to take antibacterial medication for prevention or treatment of a basic condition, such as an infection diagnosed before surgery, serious/complicated heart/pulmonary conditions, and diabetes; (2) the operation time exceeds 3 hours or bleeding exceeds more than 1500 mL; and (3) additional neck lymph node resection is required for malignant thyroid tumor.

## 5. Conclusions

Thyroidectomy involves a small incision classified as type-I incision, short duration, and minor hemorrhage. Given that the procedure is performed under stringent conditions of sterility and hemostasis, preventive antibacterial therapy for incision infection may not be required.

## Figures and Tables

**Table 1:** Patients' characteristics (total number of patients).

Parameter	Number (*n* = 116)
Sex (*n* = 1116)	(*n* = 1116)
Male	212
Female	954
Age (yrs) (*n* = 1116)	(*n* = 1116)
11–20	29
21–30	256
31–40	372
41–50	233
51–60	208
61–70	60
71–80	9
Pathology (*n* = 1116)	(*n* = 1116)
Nodular goiter	916
Nodular papillary hyperplasia	114
Graves' disease	45
Thyroid papillary carcinoma	71
Hashimoto's disease	14
Medullary carcinoma	2
Parathyroidoma	4
Preoperative disease problems (*n* = 67)	(*n* = 67)
Type 1 diabetes mellitus	32
Hypertension	34
Infiltrative pulmonary tuberculosis	1

**Table 2:** Patients' data regarding anesthesia, surgical procedure, duration of surgery, and postoperative complications.

Parameter	*n* (%)
Anesthesia (total cases = 1166)	
Tracheal intubation general anesthesia	1003 (86.0)
Cervical plexus anesthesia	163 (14.0)
Surgical procedure (*n* = 1166)	(*n* = 1166)
Unilateral total or partial thyroid lobectomy	793 (68.0)
Single lobectomy plus partial resection of opposite lobe	78 (6.7)
Bilateral partial thyroidectomy	250 (21.4)
Double lobectomy	34 (2.9)
One-sided cervical lymph node resection for papillary carcinoma	6 (0.5)
Bilateral cervical lymph node resection for papillary carcinoma	1 (0.1)
Resection of parathyroidoma	4 (0.3)
Duration of surgery (min) (*n* = 1166)	
20–69	418 (35.8)
70–120	658 (56.4)
130–170	65 (5.6)
180–230	18 (1.5)
240–330	7 (0.6)
Average operation time (min)	80.6 ± 4.87
Postoperative complications (*n* = 15)	
Incision infection	1 (0.1)
Bleeding after 24 h of surgery	5 (0.5)
Laryngeal nerve paralysis	6 (0.6)
Hypoparathyroidism	2 (0.2)
Allergic dermatitis exfoliativa caused by sodium phenobarbital	1 (0.1)
